# Associations of Physical Activity, Sedentary Behavior, and Handgrip Strength with Clinical Characteristics in Women with Rheumatoid Arthritis from Western Mexico

**DOI:** 10.3390/epidemiologia7050139

**Published:** 2026-10-07

**Authors:** Daniel Alejandro Salazar-Gutiérrez, José Francisco Muñoz-Valle, Francisco González-Rodríguez, Sergio Cerpa-Cruz, Ana Clarissa Macías-Martínez, Ilce Valeria Román-Fernández, Jorge Hernández-Bello, Eric Genaro Salmerón-Bárcenas, Samuel García-Arellano, Christian Johana Baños-Hernández

**Affiliations:** 1 Universidad de Guadalajara, Centro Universitario de Ciencias de la Salud, Instituto de Investigación en Ciencias Biomédicas, Guadalajara 44340, Jalisco, Mexico; daniel.salazar1466@alumnos.udg.mx (D.A.S.-G.); drjosefranciscomv@cucs.udg.mx (J.F.M.-V.); francisco.gonzalez7838@alumnos.udg.mx (F.G.-R.); ilce.roman3356@academicos.udg.mx (I.V.R.-F.); jorge.hernandez@academicos.udg.mx (J.H.-B.); 2 Servicio de Reumatología, O.P.D. Hospital Civil de Guadalajara “Fray Antonio Alcalde”, Guadalajara 44280, Jalisco, Mexico; sergio.cerpa@academicos.udg.mx (S.C.-C.); ana.macias5143@alumnos.udg.mx (A.C.M.-M.); 3 Departamento de Biomedicina Molecular, Centro de Investigación y de Estudios Avanzados del Instituto Politécnico Nacional, Mexico City 07360, Estado de Mexico, Mexico; eric.salmeron@cinvestav.mx

**Keywords:** rheumatoid arthritis, sedentary behavior, physical activity, handgrip strength, disability

## Abstract

Background: Rheumatoid arthritis (RA) is a chronic autoimmune disease that affects synovial joints and leads to functional impairment. Modifiable factors such as physical activity (PA), sedentary behavior (SB), and handgrip strength (HGS) may influence clinical and functional outcomes; however, these relationships have been insufficiently studied in Mexican women with RA. Therefore, this study aimed to examine the associations of PA, SB, and HGS, with clinical characteristics in women with RA from western Mexico. Methods: PA and SB were assessed using the short form of the International Physical Activity Questionnaire (IPAQ-S). Disability was evaluated using the Health Assessment Questionnaire Disability Index (HAQ-DI), HGS was measured by dynamometry, and disease activity was assessed using the Disease Activity Score in 28 joints based on C-reactive protein (DAS28-CRP). Inflammatory and serological markers (CRP, RF, anti-CCP, and ESR) were determined using standard analytical methods. Results: The study included 58 women with RA, with a mean DAS28-CRP score of 3.25 ± 1.16. PA was inversely associated with CRP levels (β = –0.312, *p* = 0.023), with higher CRP concentrations observed among patients with low PA levels. SB was positively associated with HAQ-DI scores (β = 0.345, *p* = 0.008) and DAS28-CRP scores (β = 0.420, *p* = 0.001). HGS was inversely associated with DAS28-CRP (β = –0.323, *p* = 0.04) and HAQ-DI (β = –0.521, *p* = 0.0004), independent of age and body mass index. Conclusions: In conclusion, lower PA levels were associated with higher CRP concentrations, whereas lower HGS and greater SB were associated with greater disease activity and disability in women with RA.

## 1. Introduction

Rheumatoid arthritis (RA) is a chronic autoimmune inflammatory disease that primarily affects the synovial joints, leading to cartilage degradation, bone damage, functional limitations, reduced quality of life, and disability [1]. The global prevalence of RA is estimated to be less than 1%, whereas in Mexico approximately 1.6% of the population is affected by the disease [2,3]. Although RA can develop at any stage of life, the highest prevalence is observed among individuals aged 55–69 years [2]. RA has been associated with several comorbidities, including cardiovascular disease (CVD) [4], metabolic syndrome [5], and sarcopenia, which is characterized by a progressive decline in muscle mass and strength that significantly affects physical function [6].

Physical activity (PA) encompasses any bodily movement produced by skeletal muscles that results in energy expenditure above resting levels [7]. Because of its beneficial effects on multiple aspects of health, PA is considered an important component of the comprehensive management of several chronic diseases [8]. In 2018, the European Alliance of Associations for Rheumatology (EULAR) recommended incorporating PA into the standard treatment of patients with inflammatory arthritis and osteoarthritis [9]. In individuals with RA, physical function is typically 24–34% lower than that observed in healthy controls [10]. Although the effects of PA on inflammation in RA remain inconclusive, beneficial effects have been reported for quality of life, as measured by the Health Assessment Questionnaire Disability Index (HAQ-DI), as well as for clinical parameters associated with disease progression [11,12]. Despite these benefits, a study conducted in Latin America reported that nearly 45% of individuals with RA did not meet the minimum levels of PA recommended by the World Health Organization (WHO), reflecting a high prevalence of physical inactivity and sedentary behavior in this population [13].

Sedentary behavior (SB) is defined as any waking behavior characterized by an energy expenditure of ≤1.5 metabolic equivalents (METs) while in a sitting, reclining, or lying posture and has been associated with an increased risk of CVD [14]. Several studies have reported that individuals with RA engage in higher levels of SB than the general population [15,16]. A bidirectional relationship has been proposed between sedentary behavior, inflammation, and disease progression. Specifically, SB has been associated with elevated inflammatory markers of physical activity levels. Conversely, the chronic inflammation characteristic of RA may exacerbate symptoms such as pain and stiffness, thereby promoting sedentary behavior and perpetuating this cycle. Collectively, these mechanisms may contribute to the development of associated comorbidities, particularly CVD [17].

Handgrip strength (HGS), defined as the ability of the hand to generate force during gripping, is widely used in both clinical and research settings because it is strongly associated with overall muscle strength and physical function, making it a valuable marker of health status, particularly in older adults [18]. In individuals with RA, HGS has been inversely associated with disease activity and joint damage, such that greater strength is associated with lower Disease Activity Score in 28 joints (DAS28) values and less joint damage detected by ultrasound imaging [19,20]. Furthermore, HGS has shown strong associations with functional outcomes, including disability and quality of life [21,22].

Studies examining the relationship between PA and disease activity in RA have produced inconsistent findings [23,24]. Furthermore, no study to date has evaluated the relationship among PA, SB, HGS, and clinical characteristics in women with RA from western Mexico. Therefore, the aim of this study was to analyze the associations among PA, SB, HGS, and clinical characteristics in women with RA from western Mexico.

## 2. Materials and Methods

### 2.1. Study Participants

Patients were recruited from the Rheumatology Department of the Hospital Civil de Guadalajara “Fray Antonio Alcalde” in Jalisco, Mexico, between February 2025 and February 2026. Participants met the 2010 American College of Rheumatology/European Alliance of Associations for Rheumatology (ACR/EULAR) classification criteria for RA. All participants provided written informed consent prior to enrollment. Patients with overlapping rheumatic or autoimmune diseases and those receiving biologic therapies were excluded.

Based on the effect size of a correlation between HGS and DAS-28 (Pearson’s r = −0.412) [19], a two-sided correlation analysis with α = 0.05 and 80% statistical power would require approximately 41 participants. The present study used consecutive non-probability sampling, and the final sample size was determined by the number of eligible patients recruited during the predefined study period. A total of 58 women with RA were ultimately included, exceeding the estimated sample size required to detect a correlation of similar magnitude.

### 2.2. Laboratory Tests

CRP and RF were quantified using turbidimetric assays (BioSystem, Cat. 13927 and 31922, respectively, Barcelona, Spain) on a Mindray BS-120 Chemistry Analyzer (Mindray, Shenzhen, China) according to the manufacturer’s instructions. The analytical validity of immunoturbidimetric approaches for the quantitative determination of CRP and RF has been previously established [25,26]. In addition, the same analytical approach and instrumentation have previously been used by our research group in patients with RA [27]. Patients with RF levels > 20 IU/mL were classified as RF-positive. Anti-cyclic citrullinated peptide antibodies (anti-CCP) were assessed using an enzyme-linked immunosorbent assay (ELISA; EUROIMMUN, Cat. No. EA 1505-9601 G, Lübeck, Germany) according to the manufacturer’s instructions. The lower detection limit of the assay was 0.3 U/mL, and values > 5 U/mL were considered positive. Erythrocyte sedimentation rate (ESR) was determined using the Wintrobe method and expressed as mm/h.

### 2.3. Disease Activity Assessment

Disease activity was assessed using the Disease Activity Score in 28 joints based on C-reactive protein (DAS28-CRP), which incorporates the patient global assessment (PGA), tender joint count (TJC), swollen joint count (SJC), and CRP concentration. Both the continuous DAS28-CRP score and its clinical classification were considered for analysis. Disease activity categories were defined according to EULAR criteria as high disease activity (≥5.1), moderate disease activity (≥3.2 to <5.1), low disease activity (≥2.6 to <3.2), and remission (<2.6).

Disability was assessed using the Spanish-translated and culturally adapted version of the Health Assessment Questionnaire Disability Index (HAQ-DI) [28]. Scores range from 0 to 3, with higher scores indicating greater disability. Disability was categorized as no disability (<0.25), mild disability (0.25–0.99), moderate disability (1.00–1.99), and severe disability (≥2.00).

### 2.4. Handgrip Strength Assessment

HGS was measured using a Jamar hydraulic dynamometer with the handle set at the second position, following the protocol described by Mathiowetz et al. [29]. Participants were seated with the shoulder adducted and neutrally rotated, the elbow flexed at 90°, and the wrist in a neutral position (0° of flexion). Only the dominant hand was evaluated. Although the original protocol recommends using the mean of three trials, two measurements were initially obtained to minimize discomfort, with a one-minute rest interval between trials. If the difference between measurements was ≤3 kg, the higher value was recorded. Otherwise, a third measurement was obtained, and the highest value of the three trials was used for analysis. Participants were instructed to exert maximal grip force during each trial, and standardized verbal encouragement was provided during testing. Assessments were performed at approximately the same time of day whenever possible. If a participant reported pain severe enough to interfere with test performance, the assessment was postponed, omitted, or discontinued.

### 2.5. Assessment of Physical Activity and Sedentary Behavior

PA was assessed using the short version of the International Physical Activity Questionnaire (IPAQ-S), translated and validated for Spanish-speaking populations [30], and administered through a face-to-face interview. The questionnaire consists of seven items that assess PA performed during the previous seven days and classifies activities into three categories: walking, moderate-intensity activity, and vigorous-intensity activity.

The IPAQ-S assigns standard metabolic equivalent (MET) values to each activity category, with walking corresponding to 3.3 METs, moderate-intensity activity to 4.0 METs, and vigorous-intensity activity to 8.0 s METs. In this study, PA was expressed as MET-minutes per week (MET-min/week), calculated by multiplying the corresponding MET value by the duration of activity in minutes per day and the number of days per week. Total PA was calculated as the sum of the MET-min/week scores for walking, moderate-intensity activity, and vigorous-intensity activity.

Based on the IPAQ scoring protocol, participants were classified into three PA categories: low, moderate, and high. A high level of PA was defined as engaging in vigorous-intensity activity on at least 3 days per week and accumulating a minimum of 1500 MET-min/week, or engaging in any combination of walking, moderate-intensity activity, or vigorous-intensity activity on at least 7 days per week and accumulating a minimum of 3000 MET-min/week. A moderate level of PA was defined as engaging in vigorous-intensity activity on at least 3 days per week for a minimum of 20 min/day, engaging in moderate-intensity activity and/or walking on at least 5 days per week for a minimum of 30 min/day; or engaging in any combination of walking, moderate-intensity activity, or vigorous-intensity activity on at least 5 days per week and accumulating a minimum of 600 MET-min/week. Participants who did not meet any of these criteria were classified as having a low level of PA.

The IPAQ-S also includes an item assessing SB, which records the total amount of time spent sitting on a typical day. This measure includes time spent sitting at work, at home, during transportation, and during leisure activities. In this study, total daily sitting time, expressed in minutes, was used as a continuous measure of SB.

### 2.6. Statistical Analysis

Data distribution was assessed using the Shapiro–Wilk test. Continuous variables are reported as mean ± standard deviation (SD) for normally distributed data and as median with interquartile range (IQR; 25th–75th percentile) for non-normally distributed data. Categorical variables are presented as frequencies and percentages.

Associations between variables were evaluated using Spearman’s rank correlation coefficient. Comparisons among physical activity categories were performed using the Kruskal–Wallis test, followed by Dunn’s post hoc test. Simple linear regression analyses were conducted to evaluate associations between variables, and multiple linear regression models were used to adjust for potential confounding variables selected based on their clinical and theoretical relevance. Age and BMI were included as covariates in models evaluating the association of HGS with clinical outcomes, whereas disability was included as a covariate in the model evaluating the association between SB and disease activity.

Linear regression assumptions were assessed for all models using residual-versus-predicted plots for linearity, Q–Q plots for residual normality, and VIF and tolerance values for multicollinearity. Potentially influential observations were evaluated using standardized and studentized deleted residuals, leverage, and Cook’s distance. A two-sided *p*-value ≤ 0.05 was considered statistically significant. All analyses were performed using GraphPad Prism version 8.0 (GraphPad Software, San Diego, CA, USA) and IBM SPSS Statistics version 30.0 (IBM Corp., Armonk, NY, USA).

## 3. Results

### 3.1. Clinical and Demographic Characteristics

A total of 58 women with RA were included in the study. The median age was 50 years (IQR, 39–57 years). Based on self-reported height and weight, the mean BMI was 27.82 ± 5.74 kg/m^2^. The median PA level was 1188 MET-min/week (IQR, 615–2358). The mean DAS28-CRP score was 3.25 ± 1.16, with 43.1% (n = 25) of participants classified as having moderate disease activity. The median HAQ-DI score was 0.94 (IQR, 0.5–1.63), and 36.2% (n = 21) of participants were classified as having mild disability. Detailed clinical and demographic characteristics are presented in Table 1.

### 3.2. Correlation of Physical Activity, Sedentary Behavior, and Handgrip Strength with Clinical Characteristics

A significant and medium inverse correlation was observed between PA and CRP levels (rho = −0.32, *p* = 0.02). No significant correlations were identified between PA and other clinical variables. SB was positively correlated with DAS28-CRP scores (rho = 0.36, *p* = 0.005) and HAQ-DI scores (rho = 0.35, *p* = 0.007). In contrast, HGS was inversely correlated with DAS28-CRP (rho = −0.27, *p* = 0.045) and HAQ-DI (rho = −0.38, *p* = 0.004).

**Figure 1:**
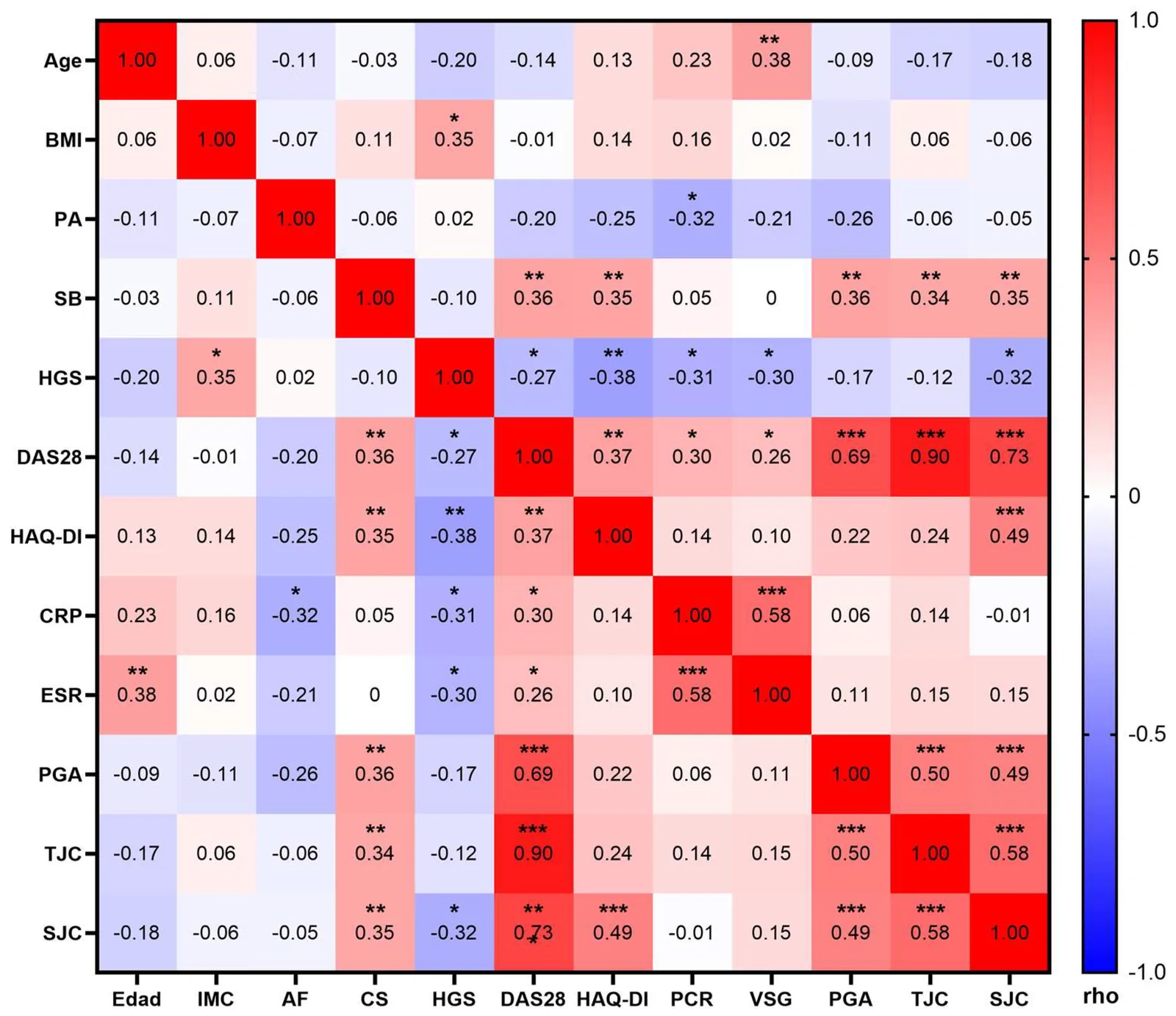
Correlation of physical activity (PA), sedentary behavior (SB), and handgrip strength (HGS) with clinical characteristics of women with RA. Correlations were assessed using Spearman’s rank correlation coefficient. *: *p* ≤ 0.05; **: *p* ≤ 0.01; *** *p* ≤ 0.001. Abbreviations: PA, physical activity; SB, sedentary behavior; BMI, body mass index; HGS, handgrip strength; CRP, C-reactive protein; ESR, erythrocyte sedimentation rate; PGA, patient global assessment; TJC, tender joint count; SJC, swollen joint count.

These findings indicate that PA, HGS, and SB are associated with specific clinical and functional characteristics in women with RA. The correlations among all study variables are presented in Figure 1.

### 3.3. Association Between Physical Activity and Inflammation

**Figure 2:**
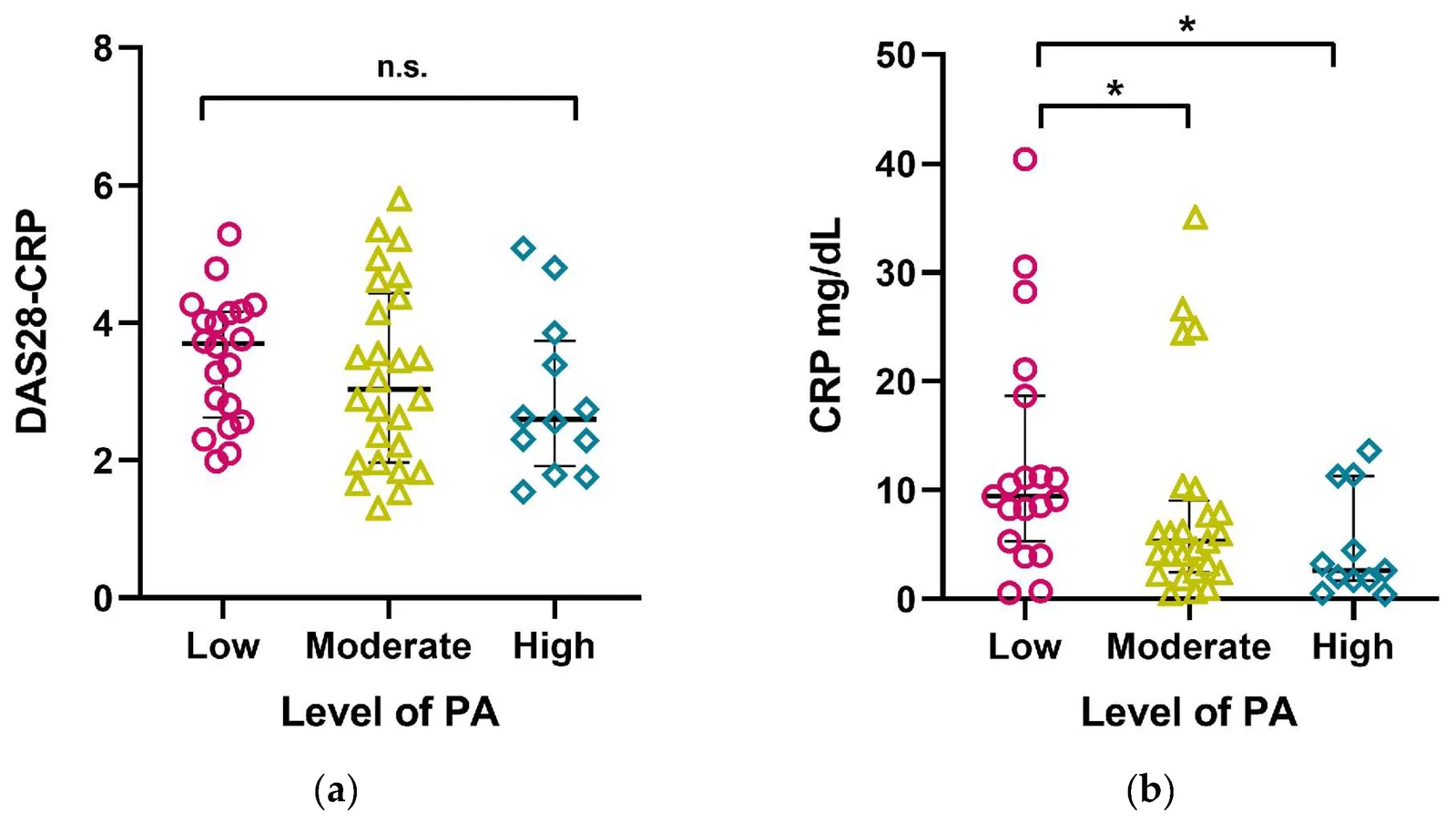
DAS28-CRP scores and CRP concentrations in women with RA stratified according to physical activity (PA) level. (**a**) DAS28-CRP scores did not differ significantly among PA categories. (**b**) Women with higher PA levels exhibited lower serum CRP concentrations than those with low PA levels. n.s.: not significant; *: *p* < 0.05. Different symbols and colors represent the physical activity groups: red ○, low physical activity; yellow △, moderate physical activity; blue ◇, high physical activity.

Although PA was not significantly associated with clinical disease activity or disability measures, a significant inverse association was observed between weekly MET-minutes and CRP concentrations (β = −0.312, r^2^ = 0.097, *p* = 0.023). Furthermore, comparison of PA categories revealed significantly lower CRP concentrations among participants with moderate (*p* = 0.0356) and high (*p* = 0.0374) PA levels compared with those reporting low PA (Figure 2).

### 3.4. Association Between Sedentary Behavior, Disability, and Disease Activity

SB was positively associated with disability (β = 0.345, r^2^ = 0.119, *p* = 0.008) and disease activity (β = 0.420, r^2^ = 0.176, *p* = 0.001). Multivariable analysis demonstrated that the association between SB and disease activity remained significant after adjustment for disability level (β = 0.317, *p* = 0.013).

Analysis of individual DAS28-CRP components revealed positive correlations between sedentary behavior and patient global assessment (PGA) (rho = 0.363, *p* = 0.005), tender joint count (TJC) (rho = 0.342, *p* = 0.009), and swollen joint count (SJC) (rho = 0.347, *p* = 0.008). No significant correlation was observed between SB and CRP concentrations (rho = 0.045, *p* = 0.738) (Figure 1).

### 3.5. Association Between Handgrip Strength, Disease Activity, and Disability

A significant inverse association was observed between HGS and HAQ-DI scores (β = −0.393, r^2^ = 0.154, *p* = 0.003). After adjustment for BMI and age, multiple linear regression analysis demonstrated that HGS remained independently associated with HAQ-DI scores (β = −0.521, *p* = 0.0004) (Table 2). In contrast, simple linear regression analysis did not demonstrate a significant association between HGS and DAS28-CRP scores (*p* = 0.087). However, after adjustment for BMI and age, a significant inverse association was observed (β = −0.323, *p* = 0.040).

## 4. Discussion

RA remains a significant clinical challenge because of its progressive and disabling nature and its higher prevalence among women than men [2]. This greater prevalence of RA in women has been attributed, at least in part, to interactions between sex hormones and the immune system, particularly during menopause, when declining estrogen levels may contribute to an increased risk and severity of the disease [31]. Therefore, identifying factors that improve quality of life, help control disease progression, and promote functional independence is essential for optimizing RA management and treatment strategies.

Although the beneficial effects of physical exercise interventions in individuals with RA have been documented, both in disease control and quality of life improvement [12], cross-sectional studies have produced inconsistent findings. Garza-Cisneros et al. [23] reported a negative association between physical exercise and DAS28 scores in Mexican patients with RA when comparing individuals who exercised with those who did not. In contrast, Ben et al. [24] found no significant relationship between clinical disease activity and PA measured using the IPAQ-S.

These findings are partially consistent with our results, as no association was observed between PA and clinical disease activity, as assessed by DAS28-CRP. However, a significant relationship was identified between PA and systemic inflammation, as measured by CRP concentrations, such that patients with higher PA levels exhibited lower CRP levels. This finding is consistent with reports from clinical trials showing that PA-based interventions in patients with RA are associated with reductions in CRP concentrations [32]. Although CRP is an important biomarker of systemic inflammation in RA, it represents only one component of the DAS28-CRP score, and changes in composite clinical indices may require structured exercise interventions implemented over longer periods. Indeed, clinical trials involving personalized aerobic and resistance exercise programs, as well as mind–body interventions, have demonstrated improvements in inflammatory markers, disease activity, and disability among patients with RA [33,34].

Although PA was not directly associated with clinical disease activity in the present study, SB was associated with both disease activity and disability. Moreover, the association between SB and disease activity remained significant after adjustment for disability level, suggesting that this relationship is not solely explained by functional impairment. Although the causal direction of this association remains unclear, interventions aimed at reducing SB have demonstrated beneficial effects in patients with RA, including reductions in inflammatory cytokines, fatigue, and pain, as well as improvements in physical function and disease control [35,36,37].

The association between SB and disease activity appears to be primarily related to the patient-reported and clinical manifestations, including PGA scores and the number of tender and swollen joints, rather than inflammatory markers such as CRP. O’Leary et al. [38] proposed that the relationship between SB and disease activity may be mediated by pain intensity, as assessed using the visual analog scale and the Widely Perceived Index. Furthermore, reductions in SB have been associated with decreases in CRP concentrations, pain, and fatigue, particularly through increased standing time [39].

SB may contribute to the maintenance and exacerbation of systemic inflammation, and in the context of RA, this could further aggravate local joint inflammation and disease activity [17]. At the same time, SB may perpetuate a pain–inactivity cycle. Increased sitting time has been associated with alterations in central pain modulation characterized by reduced inhibitory mechanisms and enhanced facilitatory pathways, ultimately increasing pain perception. This increase in pain may limit participation in PA and promote additional sedentary behavior [40]. The interaction of these mechanisms may partly explain the positive association observed between SB and disease activity.

Finally, significant associations were observed between HGS, disease activity, and disability in this population, with stronger associations evident after adjustment for age and BMI, consistent with the findings by Sheehy et al. [41]. These results are in agreement with previous studies showing that lower HGS is associated with higher disability levels, greater joint damage, and increased disease activity [19,20]. Furthermore, declines in HGS over time have been linked to reductions in physical function [42].

Reduced HGS reflects not only local hand function but also broader impairments in muscle mass and physical performance, which may translate into a diminished capacity to perform activities of daily living [18]. Women generally exhibit lower HGS values than men, a difference that has been associated with several factors, including lower BMI. Consequently, women with RA may be particularly vulnerable to functional decline associated with muscle weakness [20,43]. Although no significant relationship was observed between HGS and PA in the present study, resistance exercise interventions have been shown to improve muscle strength in this population [44,45].

The present study has several limitations that should be acknowledged. First, its cross-sectional design precludes the establishment of causal relationships among PA, SB, HGS, and clinical characteristics in women with RA. Second, the relatively small sample size and recruitment from a single center may limit the generalizability of the findings. Additionally, some variables showed non-normal distributions and potentially atypical observations were identified during regression diagnostics, although no substantial influential observations were detected. Moreover, PA and SB were assessed using the IPAQ-S, a self-reported questionnaire that is susceptible to recall and reporting bias. Because the IPAQ-S captures PA and SB over the preceding seven days, these measures may not adequately reflect long-term behavioral patterns and may be influenced by short-term variations in PA and SB during the assessment period. Furthermore, only women were included in this study; therefore, the findings cannot be generalized to men with RA. Finally, no predefined quantitative criterion was used to determine when pain was severe enough to interfere with test performance, introducing some subjectivity into this decision.

## 5. Conclusions

In women with RA, lower handgrip strength, independent of age and BMI, was associated with greater disability. In addition, lower levels of physical activity were associated with higher CRP concentrations, whereas greater sedentary behavior was associated with higher disease activity, independent of disability status. Collectively, these findings suggest that physical activity, muscle strength, and sedentary behavior may represent factors associated with clinical and functional outcomes in women with RA. However, given the cross-sectional nature of the present study, longitudinal and intervention studies are needed to further investigate these associations and their potential implications for the comprehensive management of patients with RA.

## Figures and Tables

**Table 1:** Clinical and Demographic Characteristics of Women with RA.

Characteristics	RA (58)
Demographic and anthropometric	
Age, years (IQR)	50 (38–57)
Height, m (±SD)	1.57 (±0.7)
Weight, kg (±SD)	68.68 (±14.74)
BMI, kg/m^2^ (±SD)	27.82 (±5.74)
Handgrip strength, kg (IQR)	16 (10–22)
Clinical evaluation	
Disease evolution, years (IQR)	5.5 (2–13.25)
DAS28-CRP score (±SD)	3.25 (±1.16)
DAS28-CRP category, % (n)	
Remission	34.5 (20)
Low activity	15.5 (9)
Moderate activity	43.1 (25)
High activity	6.9 (4)
HAQ-DI score (IQR)	0.94 (0.5–1.63)
HAQ-DI disability category, % (n)	
No disability	13.8 (9)
Mild disability	36.2 (21)
Moderate disability	32.8 (19)
Severe disability	17.2 (10)
Inflammatory and serological markers	
ESR, mm/h (±SD)	34.43 (±13.29)
CRP, mg/dL (IQR)	6.06 (2.56–11.14)
RF, IU/mL (IQR)	84.15 (22.24–150.4)
RF status, % (n)	
Negative (<20 IU/mL)	22 (13)
Low positive (20–59.9 IU/mL)	17 (10)
High positive (≥60 IU/mL)	61 (35)
Anti-CCP, U/mL (IQR)	91.17 (18.2–189.7)
Anti-CCP status, % (n)	
Negative (<5 U/mL)	9 (5)
Low positive (5.1–14.9 U/mL)	12 (7)
High positive (≥15 U/mL)	79 (46)
PA	
MET-min/week (IQR)	1188 (615–2358)
PA level, % (n)	
Low	34 (20)
Moderate	45 (26)
High	21 (12)
SB, min (IQR)	180 (142–300)
Drug treatment	
NSAID use, % (n)	71 (41)
DMARDs, % (n)	
Methotrexate	76 (45)
Hydroxychloroquine	38 (22)
Sulfasalazine	67 (39)
Leflunomide	14 (8)

Note: RA: rheumatoid arthritis; SD, standard deviation; IQR, interquartile range; BMI, body mass index; DAS28-CRP, disease activity score in 28 joints based on C-reactive protein; HAQ-DI, Health Assessment Questionnaire Disability Index; ESR, erythrocyte sedimentation rate; CRP, C-reactive protein; RF, rheumatoid factor; anti-CCP, anti-cyclic citrullinated peptide antibodies; PA, physical activity; MET-min/week, metabolic equivalent minutes per week; SB, sedentary behavior; DMARDs; disease-modifying antirheumatic drugs; NSAIDs; nonsteroidal anti-inflammatory drugs.

**Table 2:** Multiple Linear Regression Analysis of Handgrip Strength (HGS) and Clinical Characteristics.

	Clinical Activity (DAS28-CRP)		Disability (HAQ-DI)	
	β [95% CI]	*p*	β [95% CI]	*p*
Handgrip strength	**−0.323 [−0.094, −0.002]**	**0.04**	**−0.521 [−0.075, −0.023]**	**<0.001**
BMI	0.190 [−0.02, 0.089]	0.21	**0.337 [0.007, 0.069]**	**0.016**
Age	−0.207 [−0.055, 0.009]	0.16	−0.004 [−0.018, 0.018]	0.973

Note: HGS, handgrip strength; DAS28-CRP, disease activity score in 28 joints based on C-reactive protein; HAQ-DI, Health Assessment Questionnaire Disability Index; β, regression coefficient; CI, confidence interval; BMI, body mass index. *p*-values were obtained from multiple linear regression analyses. Statistically significant associations are shown in bold.

## Data Availability

The data supporting the findings of this study are available from the corresponding author (C.J.B.-H.) upon reasonable request.

## Abbreviations

The following abbreviations are used in this manuscript:

RA	Rheumatoid arthritis
PA	Physical activity
SB	Sedentary behavior
HGS	Handgrip strength
IPAQ-S	Short Form of the International Physical Activity Questionnaire
HAQ-DI	Health Assessment Questionnaire Disability Index
DAS28	Disease activity score in 28 joints
CRP	C-reactive protein
RF	Rheumatoid factor
Anti-CCP	Anti-cyclic citrullinated peptide antibodies
ESR	Erythrocyte sedimentation rate
CVD	Cardiovascular disease
EULAR	European Alliance of Associations for Rheumatology
MET	Metabolic equivalents
PGA	Patient global assessment
TJC	Tender joint count
SJC	Swollen joint count
IQR	Interquartile range
SD	Standard deviation
BMI	Body mass index
NSAIDs	Nonsteroidal anti-inflammatory drugs
DMARDs	Disease-modifying antirheumatic drugs
ACR	American College of Rheumatology
ELISA	Enzyme-linked immunosorbent assay
MET-min/week	Metabolic equivalent minutes per week

## References

[1] Aletaha D., Smolen J.S. (2018). Diagnosis and Management of Rheumatoid Arthritis: A Review. JAMA.

[2] Ma Y., Chen H., Lv W., Wei S., Zou Y., Li R., Wang J., She W., Yuan L., Tao J. (2025). Global, regional and national burden of rheumatoid arthritis from 1990 to 2021, with projections of incidence to 2050: A systematic and comprehensive analysis of the Global Burden of Disease study 2021. Biomark. Res..

[3] Moreno-Montoya J., Alvarez-Nemegyei J., Sanin L.H., Pérez-Barbosa L., Trejo-Valdivia B., Santana N., Goycochea-Robles M.V., Cardiel M.H., Riega-Torres J., Maradiaga M. (2015). Association of regional and cultural factors with the prevalence of rheumatoid arthritis in the Mexican population: A multilevel analysis. JCR J. Clin. Rheumatol..

[4] Weber B.N., Giles J.T., Liao K.P. (2023). Shared inflammatory pathways of rheumatoid arthritis and atherosclerotic cardiovascular disease. Nat. Rev. Rheumatol..

[5] Cai W., Tang X., Pang M. (2022). Prevalence of Metabolic Syndrome in Patients with Rheumatoid Arthritis: An Updated Systematic Review and Meta-Analysis. Front. Med..

[6] Khoja S.S., Moore C.G., Goodpaster B.H., Delitto A., Piva S.R. (2018). Skeletal Muscle Fat and Its Association with Physical Function in Rheumatoid Arthritis. Arthritis Care Res..

[7] Caspersen C.J., Powell K.E., Christenson G.M. (1985). Physical activity, exercise, and physical fitness: Definitions and distinctions for health-related research. Public Health Rep..

[8] Rao P., Belanger M.J., Robbins J.M. (2022). Exercise, physical activity, and cardiometabolic health: Insights into the prevention and treatment of cardiometabolic diseases. Cardiol. Rev..

[9] Rausch Osthoff A.K., Niedermann K., Braun J., Adams J., Brodin N., Dagfinrud H., Duruoz T., Esbensen B.A., Günther K.-P., Hurkmans E. (2018). 2018 EULAR recommendations for physical activity in people with inflammatory arthritis and osteoarthritis. Ann. Rheum. Dis..

[10] Lemmey A.B., Wilkinson T.J., Clayton R.J., Sheikh F., Whale J., Jones H.S., Ahmad Y.A., Chitale S., Jones J.G., Maddison P.J. (2016). Tight control of disease activity fails to improve body composition or physical function in rheumatoid arthritis patients. Rheumatology.

[11] Björk M., Dragioti E., Alexandersson H., Esbensen B.A., Boström C., Friden C., Hjalmarsson S., Hörnberg K., Kjeken I., Regardt M. (2022). Inflammatory arthritis and the effect of physical activity on quality of life and self-reported function: A systematic review and meta-analysis. Arthritis Care Res..

[12] Hu H., Xu A., Gao C., Wang Z., Wu X. (2021). The effect of physical exercise on rheumatoid arthritis: An overview of systematic reviews and meta-analysis. J. Adv. Nurs..

[13] Franco M., Ferreira-Gaona M.I., Díaz-Reissner C., Avila-Pedretti G., Mazzoleni J. (2021). Physical activity in patients with rheumatoid arthritis: Cross sectional study. Rev. Paraguaya Reumatol..

[14] Tremblay M.S., Aubert S., Barnes J.D., Saunders T.J., Carson V., Latimer-Cheung A.E., Chastin S.F., Altenburg T.M., Chinapaw M.J. (2017). Sedentary Behavior Research Network (SBRN)–terminology consensus project process and outcome. Int. J. Behav. Nutr. Phys. Act..

[15] Barker J., Smith Byrne K., Doherty A., Foster C., Rahimi K., Ramakrishnan R., Woodward M., Dwyer T. (2019). Physical activity of UK adults with chronic disease: Cross-sectional analysis of accelerometer-measured physical activity in 96 706 UK Biobank participants. Int. J. Epidemiol..

[16] Prioreschi A., Hodkinson B., Avidon I., Tikly M., McVeigh J.A. (2013). The clinical utility of accelerometry in patients with rheumatoid arthritis. Rheumatology.

[17] Fenton S.A., Veldhuijzen van Zanten J.J., Duda J.L., Metsios G.S., Kitas G.D. (2018). Sedentary behavior in rheumatoid arthritis: Definition, measurement and implications for health. Rheumatology.

[18] Bohannon R.W. (2019). Grip strength: An indispensable biomarker for older adults. Clin. Interv. Aging.

[19] Palamar D., Er G., Terlemez R., Ustun I., Can G., Saridogan M. (2017). Disease activity, handgrip strengths, and hand dexterity in patients with rheumatoid arthritis. Clin. Rheumatol..

[20] Salaffi F., Carotti M., Farah S., Ceccarelli L., Di Carlo M. (2021). Handgrip strength features in rheumatoid arthritis patients assessed using an innovative cylindrical-shaped device: Relationships with demographic, anthropometric and clinical variables. J. Med. Syst..

[21] Higgins S.C., Adams J., Hughes R. (2018). Measuring hand grip strength in rheumatoid arthritis. Rheumatol. Int..

[22] Sobue Y., Suzuki M., Ohashi Y., Koshima H., Okui N., Funahashi K., Ishikawa H., Inoue H., Kojima M., Asai S. (2022). Validation of grip strength as a measure of frailty in rheumatoid arthritis. Sci. Rep..

[23] Garza-Cisneros A.N., Colunga-Pedraza I.J., Galarza-Delgado D.Á., Azpiri-López J.R., Rodriguez-Romero A.B., Balderas-Palacios M.A., Garcia-Heredia A., Guajardo-Jauregui N., Cárdenas A. (2022). AB0199 Relationship between exercise and disease activity in rheumatoid arthritis. Ann. Rheum. Dis..

[24] Tekaya A.B., Rouached L., Dhia S.B., Bouden S., Tekaya R., Mahmoud I., Saidane O., Abdelmoula L. (2024). Physical activity assessment in rheumatoid arthritis: A 120-patient cross-sectional study. Tunis. Médicale.

[25] Price C.P., Trull A.K., Berry D., Gorman E.G. (1987). Development and validation of a particle-enhanced turbidimetric immunoassay for C-reactive protein. J. Immunol. Methods.

[26] Melamies L.M., Ruutsalo M.H., Nissilä H. (1986). Evaluation of a quantitative immunoturbidimetric assay for rheumatoid factors. Clin. Chem..

[27] Baños-Hernández C.J., Navarro-Zarza J.E., Parra-Rojas I., Vázquez-Villamar M., Padilla-Gutiérrez J.R., Valle Y., Reyes-Castillo Z., Torres-Carrillo N.M., García-Arellano S., Brennan-Bourdon L.M. (2017). PADI4 polymorphisms and the functional haplotype are associated with increased rheumatoid arthritis susceptibility: A replication study in a Southern Mexican population. Hum. Immunol..

[28] Cardiel M.H., Abello-Banfi M., Ruiz-Mercado R., Alarcon-Segovia D. (1993). How to measure health status in rheumatoid arthritis in non-English speaking patients: Validation of a Spanish version of the Health Assessment Questionnaire Disability Index (Spanish HAQ-DI). Clin. Exp. Rheumatol..

[29] Mathiowetz V., Weber K., Volland G., Kashman N. (1984). Reliability and validity of grip and pinch strength evaluations. J. Hand Surg..

[30] Craig C.L., Marshall A.L., Sjöström M., Bauman A.E., Booth M.L., Ainsworth B.E., Pratt M., Ekelund U.L.F., Yngve A., Sallis J.F. (2003). International physical activity questionnaire: 12-country reliability and validity. Med. Sci. Sports Exerc..

[31] Raine C., Giles I. (2022). What is the impact of sex hormones on the pathogenesis of rheumatoid arthritis?. Front. Med..

[32] Acar A., Guzel S., Sarifakioglu B., Guzel E.C., Guzelant A.Y., Karadag C., Kiziler L. (2016). Calprotectin levels in patients with rheumatoid arthritis to assess and association with exercise treatment. Clin. Rheumatol..

[33] Stavropoulos-Kalinoglou A., Metsios G.S., Veldhuijzen van Zanten J.J., Nightingale P., Kitas G.D., Koutedakis Y. (2013). Individualised aerobic and resistance exercise training improves cardiorespiratory fitness and reduces cardiovascular risk in patients with rheumatoid arthritis. Ann. Rheum. Dis..

[34] Gautam S., Tolahunase M., Kumar U., Dada R. (2019). Impact of yoga based mind-body intervention on systemic inflammatory markers and co-morbid depression in active rheumatoid arthritis patients: A randomized controlled trial. Restor. Neurol. Neurosci..

[35] Li L.C., Xie H., Feehan L.M., Shaw C., Lu N., Ramachandran S., Wang E., Therrien S., Mucha J., Hoens A.M. (2025). Effect of digital monitoring and counselling on self-management ability in patients with rheumatoid arthritis: A randomised controlled trial. Rheumatology.

[36] Pinto A.J., Meireles K., Pecanha T., Mazzolani B.C., Smaira F.I., Rezende D., Benatti F.B., Ribeiro A.C., Pinto A.L., Lima F.R. (2021). Acute cardiometabolic effects of brief active breaks in sitting for patients with rheumatoid arthritis. Am. J. Physiol.-Endocrinol. Metab..

[37] Thomsen T., Aadahl M., Beyer N., Hetland M.L., Løppenthin K., Midtgaard J., Christensen R., Østergaard M., Jennum P.J., Esbensen B.A. (2017). The efficacy of motivational counselling and SMS reminders on daily sitting time in patients with rheumatoid arthritis: A randomised controlled trial. Ann. Rheum. Dis..

[38] O’Leary H., Larkin L., Murphy G.M., Quinn K. (2021). Relationship between pain and sedentary behavior in rheumatoid arthritis patients: A cross-sectional study. Arthritis Care Res..

[39] O’Brien C.M., Duda J.L., Kitas G.D., Veldhuijzen van Zanten J.J., Metsios G.S., Fenton S.A. (2022). Autonomous motivation to reduce sedentary behaviour is associated with less sedentary time and improved health outcomes in rheumatoid arthritis: A longitudinal study. BMC Rheumatol..

[40] Sluka K.A., Law L.F., Bement M.H. (2018). Exercise-induced pain and analgesia? Underlying mechanisms and clinical translation. Pain.

[41] Sheehy C., Gaffney K., Mukhtyar C. (2013). Standardized grip strength as an outcome measure in early rheumatoid arthritis. Scand. J. Rheumatol..

[42] Santo R.C.D.E., Baker J.F., dos Santos L.P., Silva J.M.D.S., Filippin L.I., Portes J.K.S., Brenol C.V., Chakr R.M.D.S., Xavier R.M. (2023). Changes in physical function over time in rheumatoid arthritis patients: A cohort study. PLoS ONE.

[43] Häkkinen A., Kautiainen H., Hannonen P., Ylinen J., Mäkinen H., Sokka T. (2006). Muscle strength, pain, and disease activity explain individual subdimensions of the Health Assessment Questionnaire disability index, especially in women with rheumatoid arthritis. Ann. Rheum. Dis..

[44] Pineda-Juarez J.A., Lozada-Mellado M., Hinojosa-Azaola A., Garcia-Morales J.M., Ogata-Medel M., Llorente L., Alcocer-Varela J., Orea-Tejeda A., Martín-Nares E., Castillo-Martinez L. (2022). Changes in hand grip strength and body weight after a dynamic exercise program and Mediterranean diet in women with rheumatoid arthritis: A randomized clinical trial. Physiother. Theory Pract..

[45] Azeez M., Clancy C., O’Dwyer T., Lahiff C., Wilson F., Cunnane G. (2020). Benefits of exercise in patients with rheumatoid arthritis: A randomized controlled trial of a patient-specific exercise programme. Clin. Rheumatol..

